# Temporal profiling of host transcriptome highlights time- and tissue-dependent Interferon pathway activation in NNV-infected European sea bass

**DOI:** 10.1038/s41598-025-09705-5

**Published:** 2025-07-10

**Authors:** Luca Peruzza, Giulia Dalla Rovere, Serena Ferraresso, Rafaella Franch, Daniela Bertotto, Francesco Pascoli, Gaia Bacchin, Anna Toffan, Luca Bargelloni

**Affiliations:** 1 Department of Comparative Biomedicine and Food Science, University of Padova, Viale Dell’Università 16, 35020 Legnaro, PD Italy; 2 National Reference Laboratory for Fish Diseases, Istituto Zooprofilattico Sperimentale Delle Venezie, Viale Dell’Università 10, 35020 Legnaro, PD Italy

**Keywords:** RNA-seq, Nervous Necrosis Virus, European sea bass, Interferon, Time series expression analysis, Gene expression, RNA sequencing, Immunology, Infection

## Abstract

Nervous Necrosis Virus (NNV) is a major threat to aquaculture, causing high mortality in farmed fish, including European sea bass. The genetic basis of host response to NNV has been well characterised, suggesting the potential role of interferon-induced genes in resistance to the virus, although the molecular mechanisms underlying NNV infection in sea bass are still debated. The time- and tissue-specific dynamics of gene expression is crucial for understanding host response to NNV. Here, we report on a time-course transcriptome analysis of brain and head kidney in NNV-infected bass that integrated the statistical evidence of differential expression with the information on temporal profiles (i.e. 6, 12, 24, 48, and 72 h post-infection (hpi)) compared to mock-infected controls. Results revealed substantial changes in gene expression over time, particularly for brain, with downregulation of genes involved in nervous system functions and upregulation of immune and inflammatory response genes from 24 to 48 hpi onwards, mostly associated with the interferon (IFN) response. The study highlights tissue-specific differences in the timing and magnitude of the innate immune response compared to other fish species and provides a comprehensive view of the dynamic host response, emphasizing the need for time-course studies in understanding viral pathogenesis.

## Introduction

Global marine aquaculture constitutes an important economic sector, which has seen an increased production more than 4 times since 1990^1^. Among the most widely farmed marine species, the European sea bass *Dicentrarchus labrax* is ranked 2nd in terms of annual tonnes of fish produced, according to the latest (2022) Economic Report on the EU Aquaculture and has generated a total economic value of 540 million USD in 2020 among EU countries^1^. However, the intensive farming of fish has favoured the spread of infectious diseases, which currently represent a major limitation in the productive process of these marine resources and cause important economic losses in this industry.

One of the major diseases affecting sea bass aquaculture is probably constituted by the Nervous Necrosis Virus (NNV), a single stranded, positive sense mRNA virus that causes viral encephalopathy and retinopathy (VER) or viral nervous necrosis (VNN), a disease characterised by extensive damage to brain and the central nervous system^2,3^. NNV has a neurotropic nature and infected fish show clinical signs including abnormal swimming behaviour, most often in circles. Mortality can reach 100% in infected animals, especially among juveniles which are the most susceptible life-stages^4^. This virus has a very broad range of host species^3^ and, so far, 4 different genotypes (i.e. RGNNV, SJNNV, BFNNV and TPNNV) have been isolated globally. At present the RGNNV strain seems to be the most pathogenic for the European sea bass^5,6^, with the broadest range of susceptible species^7^.

Since the first report of VNN outbreaks, a great effort has been devoted in trying to understand the molecular and physiological changes elicited by the replicating virus and the countermeasures triggered by the host to prevent the spread of the disease. In this context, transcriptomics has played a central role, allowing a genome-wide view of the host response at molecular level. In fact, several studies have employed RNA-seq to understand the effects of NNV in *in-vitro* infected cells, e.g.^8–11^, and also *in-vivo* on various species, e.g.^7,12–15^. All studies reported so far were based on “bulk” RNA-seq, although recently a single-cell approach has been adopted to characterise distinct cell populations in the mid-brain of the red spotted grouper^16^.

Following NNV infection an evident activation of the immune response and interferon (IFN) pathway has been observed in several species (e.g. ^6,8–12,16,17^), which is expected as interferon-mediated response is possibly the principal line of defence against viruses^18,19^. Quite recently, a major Quantitative Trait Locus (QTL) for resistance to VNN has been reported in the European sea bass by Delpuech, et al. ^20^, where the most significant genetic marker mapped in the proximity of a gene encoding a *interferon‑induced protein IFI6/IFI27‑like*. In the same species, a higher expression for at least three copies of *interferon‑induced protein IFI6/IFI27‑like* encoding genes was found in VNN-resistant fish^21^. However, a recent RNA-seq analysis by Lama, et al. ^15^ found a modest induction of the immune response in the brain of RGNNV infected European sea bass 24 and 72 h post infection (hpi), while observing a strong modulation of genes associated with the hypothalamic-pituitary-interrenal (HPI) axis. To reconcile all currently available evidence, the present study was set out to perform a detailed time-course analysis of the early transcriptome response of the European sea bass to RGNNV infection, to assess the extent of immune activation and its timing. RNA-seq data were obtained for five time points, namely 6, 12, 24, 48, and 72 h-post-infection (hpi). For each time point, tissue samples from brain and head-kidney either in RGNNV-challenged or mock-infected individuals (five biological replicates for each condition). Transcriptome profiles were explored through a dedicated approach for time-course data, based on functional gene annotation and temporal patterns^22^. To this end, a series of custom gene sets was implemented using comparative evidence for different vertebrate species on immune and stress response.

## Materials and methods

### Virus preparation

The virus was selected according to its role in high pathogenicity for fish. In 2009, RGNNV 283.2009 was isolated from severely affected sea bass during an outbreak in a commercial farm in the northern Adriatic Sea. The isolate was propagated on E11 cells^23^, a clone of SSN1 cell line^24^, in 150 cm^2^ tissue culture flasks.

The collected virus was subjected to titration by endpoint dilutions assays. Titres were calculated according to the Spearman–Karber formula^25^ and expressed as TCID_50_/ml.

### Experimental infection

All experiments and VNN challenge protocols were authorized by the Italian Ministry of Health (auth. 975/2016-PR). Approximately 100 fish of 13g mean weight were purchased from Valle Cà Zuliani Società Agricola srl (Pila di Porto Tolle, Rovigo, Italy) and transferred to the Istituto Zooprofilattico Sperimentale delle Venezie (IZSVe, Legnaro, Padova, Italy) experimental aquarium. At arrival, fish were distributed into two tanks of 300 L each; tanks were filled with artificial saltwater (30‰ salinity, temperature 21 ± 1 °C, oxygen 6 ppm) and exposed to artificial photoperiod (10:14 light:darkness). After an acclimation period of 14 days, 50 fish were VNN challenged through an intraperitoneal injection of 0.1 mL of viral suspension (RGNNV 283.2009, 108.30 TCID50 per mL), while 50 were mock-challenged through injection of Dulbecco’s Modified Eagle Medium (DMEM), the cell culture medium where the virus was grown. All handlings of fish were carried out under anesthesia using Tricaine (Pharmaq). At each time point (i.e. 6 h, 12 h, 24 h, 48 h or 72 h post-challenge), 5 fish per group (infected and mock) were euthanized with anesthetic overdose (Traicaine, Pharmaq) and brain and head kidney tissues were sampled for RNA sequencing. Mortality data associated with the experimental conditions used (e.g. viral strain and viral titre) is presented in a companion paper by Mukiibi et al. ^21^ with an overall mortality rate of 46.87% over 29 days.

### RNA extraction and library preparation

For sequencing and bioinformatics analyses, RNA was extracted from each of the samples using RNeasy Mini Kit (Qiagen, Hilden, Germany), according to the manufacturer’s instructions. RNA quality and concentration were checked using Agilent 2100 Bioanalyzer with RNA Nano 6000 kit (Agilent Technologies, Santa Clara, CA, USA) and only RNA samples with RNA-Integrity Number (RIN) > 7 were further processed. The cDNA libraries were constructed using QuantSeq 3′ mRNA-Seq Library Prep Kit FWD for Illumina (Lexogen GmbH, Vienna, Austria) according to the manufacturer’s recommendations. RNA extractions and library preparations were randomised, to avoid technical batch effects. Quality and concentration for each of the libraries was checked by Qubit Fluorometer (Invitrogen, Carlsbad, CA, USA) and by Agilent 2100 Bioanalyzer. All samples were pooled together and sequenced on HighSeq 4000 with a single 1 × 75 bp setup using ‘Version2’ chemistry at CRIBI facility (CRIBI; University of Padua, Italy). The sequences obtained are available in NCBI Sequence Read Archive BioProject PRJNA1200971).

For each time point (i.e. 6, 12, 24, 48 and 72 hpi) and condition (i.e. mock and NNV-infected) we collected and sequenced brain and head kidney tissues from 5 distinct animals, yielding a total of 100 RNA-seq libraries (i.e. 5 time-points × 2 conditions × 2 tissues × 5 biological replicates).

### Bioinformatic analyses

Raw reads were quality-checked using the program FastQC/v0.11.9 (https://www.bioinformatics.babraham.ac.uk/projects/fastqc/); low-quality reads and priming adaptors were removed using BBduk^26^ according to the recommendations of the developer of the sequencing kit and as detailed in Peruzza, et al. ^14^. Clean reads were then mapped on the European sea bass genome, retrieved from NCBI (https://www.ncbi.nlm.nih.gov/datasets/genome/GCF_905237075.1/) using STAR/v2.7.3a^27^. Mapping options followed recommendations from the developer of the sequencing kit (e.g. “–outFilterMismatchNoverLmax 0.1”). Gene counts were retrieved from .bam files using featureCounts/v2.0.0^28^ using the sea bass genome and annotation (in .gtf format) by including multimapping reads (options “-M” and “–fraction”) and by setting the strandness of the library (option “-s 0”). Sea bass annotation was manually edited to resolve an annotation problem that was visually discovered by comparing NCBI and Ensembl (assembly version: dlabrax2021) annotations. NCBI’s genomic scaffold NW_026136703.1 was missing the annotation of an interferon alpha-induced protein which was present however in Ensembl’s annotation (ENSEMBLID: ENSDLAG00005026832). The annotation file of NCBI in “.gtf” format was then manually edited to include the annotation of ENSDLAG00005026832.

Gene counts were then imported into Rstudio running R/v.3.6^29^ and preprocessed using the package tidyverse/v1.3.1^30^. Counts were then split by tissue and analysed separately using the following steps: the count table was filtered to remove genes with less than 5 reads in total, to remove uninformative genes which could contribute to background noise^31,32^; then counts were imported into DESeq2/v1.39^33^ with the function DESeqDataSetFromMatrix and then normalized using the “varianceStabilizingTransformation” function of the same package. Then for each combination of condition, time and tissue, normalized counts were averaged across all biological replicates using the function “avearrays” of the package limma/v3.50^34^ to comply with the requirements of the FunPat package. Averaged counts were then imported into FunPat/v0.99^22^.

Time-dependent differential expression analysis was performed with the function “SEL.TS.AREA” and by opting for an expression-level dependency of the model error built from the replicates. Subsequently gene-set based clustering and gene selection were performed according to the code provided in the FunPat vignette (by setting the threshold on adjusted p-values to 0.1) with the functions “PatternAnalysis” (setting “sizecl = 2”) and “resGSPatterns”. As functional information provided to “PatternAnalysis” different gene sets were provided (Table 1, Suppl. Table 10).

**Table 1:** List of custom gene sets used in this work.

Custom gene set name	Description	References
Core ISG	Genes upregulated by IFN across 10 different vertebrate species	Shaw et al.^39^
Murine ISG	Genes modulated by IFN-ß in mouse dendritic cell lines	Goczi et al.^41^
HPI axis	Genes encoding hormones involved in the HPI axis, modulated by NNV infection	Lama et al.^15^
Macrophage	Gene Ontologies (GOs) from zebrafish associated with macrophages	
Immune cell atlas	Marker genes defined by differential expression tests for eight major immune cell lineages	Sun et al.^37^
GO Stress response	Gene Ontology "Response to Stress" (GO:0006950)	

Heatmaps were generated using the ComplexHeatmap/v.2.14 package^35^.

Viral data was obtained by running the program VIRTUS2 (https://github.com/yyoshiaki/VIRTUS2) with default options. As viral input, the viral database from the github virdetect, which contains full genome sequences of 1894 viruses, was used (https://github.com/dmarron/virdetect/blob/master/reference/virus_unmasked.fa).

### Ethical declaration

The experimental protocol for fish challenges was designed in compliance with the Directive 2010/63/EU and the national Legislative Decree No. 26/2014. The experimental design was evaluated by the Istituto Zooprofilattico Sperimentale delle Venezie (IZSVe) Animal Welfare Body and Ethics Committee and finally approved by the Italian Ministry of Health with the authorization 975/2016-PR. All methods used in this study were in accordance with ARRIVE guidelines.

## Results

### Exploratory unsupervised analyses

In all analyses, the two tissues (i.e. brain and head kidney, hereafter “Br” and “Hk”) were processed separately. In total we generated 835,549,953 high-quality reads that were mapped to the novel version of the European sea bass genome from NCBI (i.e. GCA_905237075.1) with an average mapping success of 97.26% and 97.10% for Br and Hk, respectively (Table 2). After filtering to remove lowly expressed genes, who could contribute to background noise^31,36^, the set of analysed genes was composed of 23,379 and 23,619 genes for Br and Hk, respectively.

**Table 2:** Descriptive statistics of the RNAseq data used in this study, including the number of genes who had a significant time-dependent temporal pattern according to FunPat.

	Brain	Head kidney
Number of time points analysed	5	5
Replicates per time point	5	5
Total RNAseq libraries	50	50
Average mapping (%)	97.26%	97.10%
Total genes analysed after filtering	23,379	23,619
Total DE genes (FDR < 0.05)	3953	157

An unsupervised analysis by means of principal component analysis (PCA) revealed that the infection drove a substantial change in gene expression with time in both tissues (Suppl. Fig. 1). However, the entity of such change differed quite importantly between brain and head kidney. Along the first component of variation a clear separation of the infected samples at 48 and 72 hpi was more evident in Br than in Hk.

### Time-course and pattern analyses

Our time-course approach with FunPat identified 1,930 and 157 differentially expressed genes for Br and Hk, respectively (Suppl. Tables 1 and 2). This confirmed the pattern observed by means of PCA, where a greater change in gene expression was apparent in Br. The last two time points (48h and 72h) constituted the main driver in the separation of samples along the first component of the PCA.

**Fig. 1:**
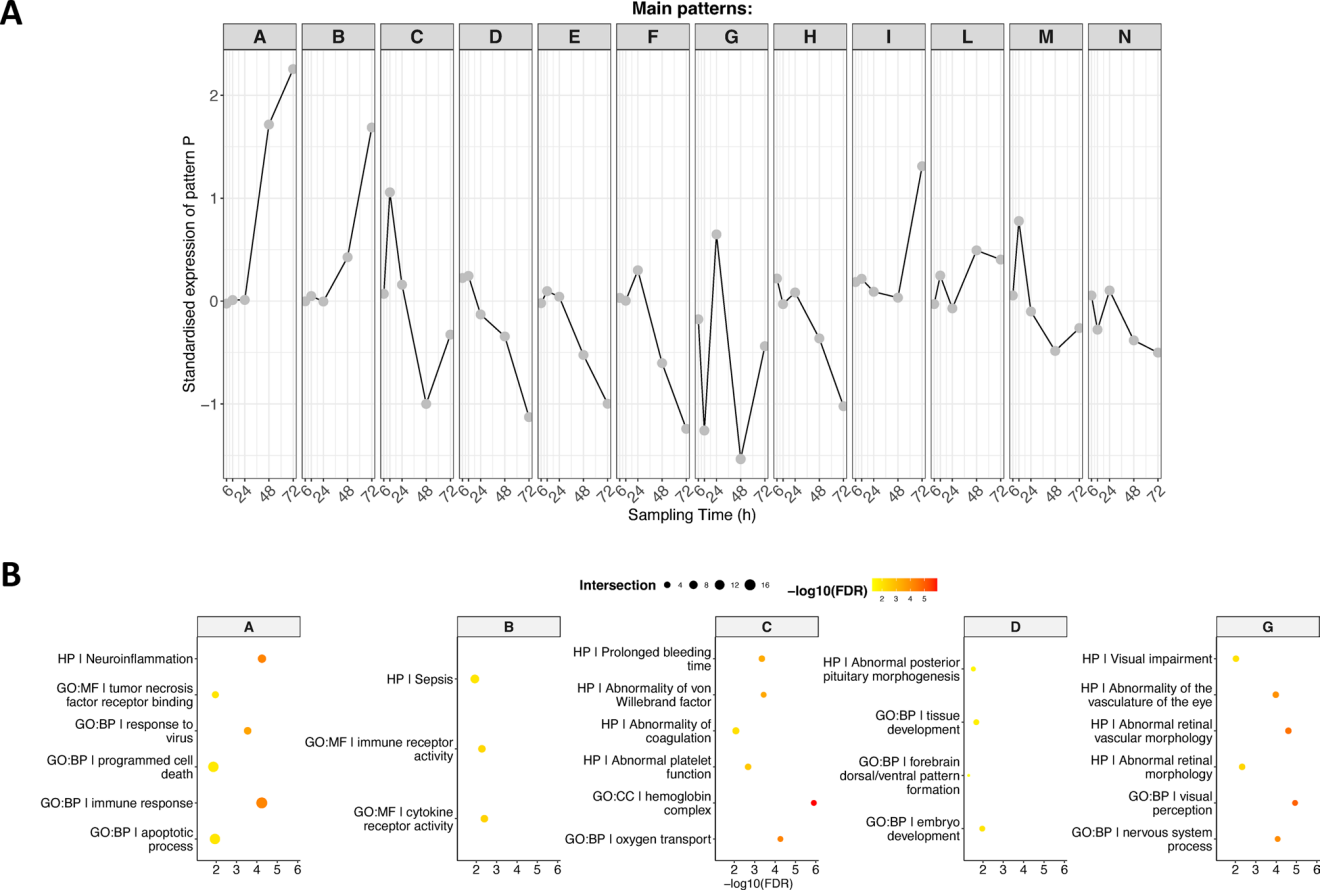
Patterns of genes showing significant differential expression with time in brain and their enriched functions. (**A**) Different gene clusters identified according to their expression profile. Grey dots indicate the average expression for a specific time point across all genes belonging to the cluster. The standardised expression is calculated by subtracting the average gene expression across control replicate samples at one time point from the average gene expression across NNV-infected replicates at the same time point. (**B**) Over-represented pathways found for each cluster of (**A**). Pathways are colour coded according the -log10 of their false discovery rate (FDR). The size of the dots is proportional to the number of differentially expressed genes belonging to the pathway.

**Fig. 2:**
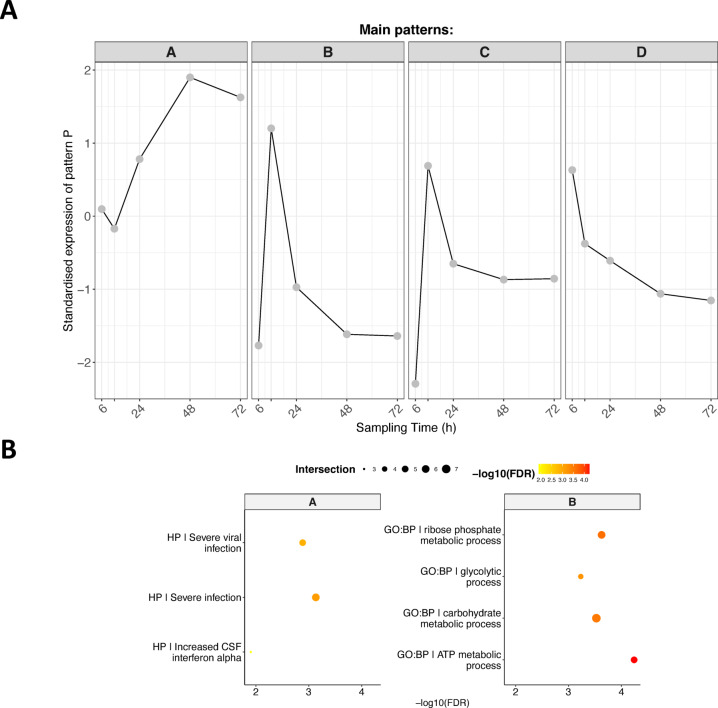
Patterns of genes showing significant differential expression with time in head kidney and their enriched functions. (**A**) Different gene clusters identified according to their expression profile. Grey dots indicate the average expression for a specific time point across all genes belonging to the cluster. The standardised expression is calculated by subtracting the average gene expression across control replicate samples at one time point from the average gene expression across NNV-infected replicates at the same time point. (**B**) Over-represented pathways found for each cluster of (**A**). Pathways are colour coded according the − log10 of their false discovery rate (FDR). The size of the dots is proportional to the number of differentially expressed genes belonging to the pathway.

Genes with a significant temporal profile were grouped by FunPat into distinct patterns based on their overall expression trajectory. For Br, we identified 12 distinct patterns (Fig. 1A, Suppl. Tables 3, 4): of these, three were characterised by a profile of expression that was increasing with time in NNV infected animals (i.e. A, B and I), five showed a decreasing expression with time (i.e. D, E, F, H, M) and four showed opposite changes in expression levels between consecutive time points (i.e. C, G, L, N). Clusters of genes displaying increasing expression with time were enriched by immunity and inflammatory processes (e.g. “Neuroinflammation”, “Response to virus” or “immune receptor activity”, Fig. 1B), clusters with decreasing expression with time were enriched by processes suggesting an impact of NNV on tissue homeostasis and maintenance (e.g. the significant enrichment of the pathway “tissue development” and the decreased expression of “*smo”*—smoothened, frizzled class receptor—an important gene involved in tissue homeostasis, repair and maintenance of stem cell populations, particularly in the brain), whereas clusters with sharp changes between consecutive points were enriched by processes impacting blood coagulation and platelet function (e.g. “prolonged bleeding time”, “abnormal platelet function”) and by alterations associated with the eye (e.g. “visual impairment/perception”). For Hk, differentially expressed genes were grouped in four clusters (Fig. 2A, Suppl. Tables 5, 6); of these, one was characterised by increasing expression with time, two were characterised by a sharp peak at 12 hpi followed by a decrease in expression at later time points and one by an almost linear decrease in expression with time. Enrichment analysis revealed that the first group was enriched with infection-related biological processes (e.g. “severe viral infection”, Fig. 2B), while the second was enriched with metabolic functions (e.g. “ATP metabolic process”, “carbohydrate metabolic process”).

### Time-course with custom gene sets

**Fig. 3:**
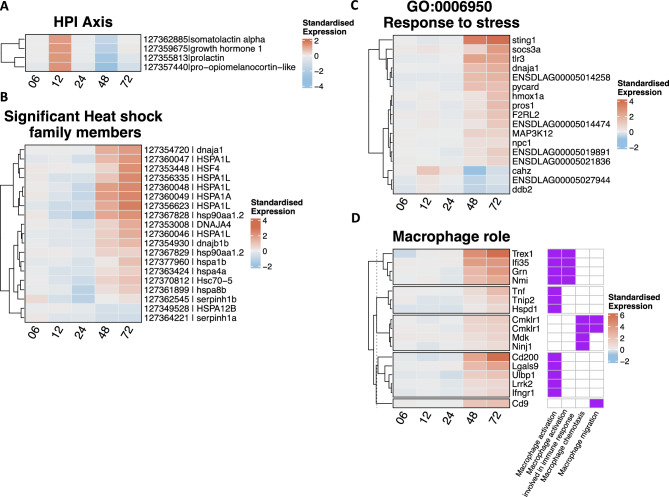
Heatmap of gene sets showing a significant time-dependent expression profile in brain tissue. The standardised expression is calculated by subtracting the average gene expression across control replicate samples at one time point from the average gene expression across NNV-infected replicates at the same time point after previous log_2_ transformation of the whole expression dataset. Please note that each heatmap is expressed on a log_2_ scale. (**A**) Genes of the gene set “HPI axis”; (**B**) Genes belonging to the heat shock family members; (**C**) Genes included in the gene ontology “response to stress”; (**D**) genes of the “Macrophage” gene set. This heatmap is associated with a presence/absence heatmap depicting the membership of each gene to a specific pathway(s) with a purple square. Pathway names are reported at the bottom of the heatmap.

The list of differentially expressed genes was further combined by FunPat with custom gene sets to reveal gene set-specific patterns. We initially tested the stress response reported by Lama et al. ^15^, who identified differentially expressed genes (DEGs) of the HPI axis in sea bass following NNV infection. We found that four genes encoding pituitary hormones (i.e. *somatolactin alpha*, *growth hormone 1*, *prolactin* and *pro-opiomelanocortin*) had a significant temporal pattern in Br, with an up-regulation at 12 hpi and a notable down-regulation at 48 hpi (Fig. 3A, Suppl. Table 7). No significant temporal pattern for the same gene set was found in Hk. In NNV infected animals, 19 genes belonging to the Heat Shock Protein (HSP) family showed a significant temporal pattern of expression in Br (Fig. 3B, Suppl. Table 1), while only one was present in Hk (Suppl. Table 2). The pattern of expression of those HSPs genes in Br revealed an important up-regulation only later during the infection (i.e. from 48 hpi onward) while between 12 and 24 hpi most HSPs genes were down-regulated. To further confirm the induction of stress response in NNV-infected brain we tested the GO “response to stress” (GO:0006950) and found 17 genes belonging to this gene set whose expression significantly increased with time in infected seabass (Fig. 3C, Suppl. Table 7). It should be noted that at least two genes in this set are directly involved in antiviral response, as STING1 is the direct effector of the DNA/RNA sensor cGAS and TLR3 acts as receptor for dsRNA in endosomes. In Hk the GO “response to stress” was not significantly enriched.

We then screened for molecular signatures characteristics of specific types of immune cells by taking advantage of a recent immune cell atlas from Sun, et al. ^37^ and found that the gene signature of mononuclear phagocyte (MPs, i.e. macrophages), endothelial cells and T cells could be detected and was positively associated with the course of infection in Br (Suppl. Fig. 2, Suppl. Table 7) and, to a lesser extent, in Hk (Suppl. Table 8). In particular, we found eleven genes involved in “macrophage activation” and four genes involved in “macrophage chemotaxis and migration” that showed a clear temporal pattern of increasing expression with a peak at 72 hpi, including the exonuclease *Trex1*, which is induced in macrophages in response to proinflammatory stimuli and acts to restrain the proinflammatory activation^38^ (Fig. 3D, Suppl. Table 7).

**Fig. 4:**
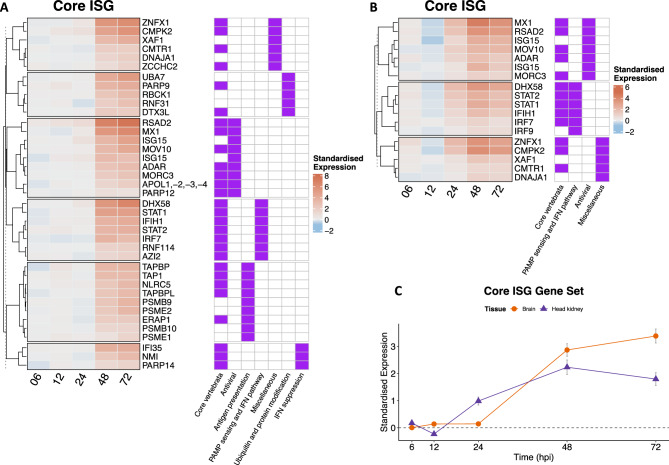
Heatmap of gene sets showing a significant time-dependent expression profile of the “core ISG” gene set in (**A**) brain tissue and (**B**) head kidney. The standardised expression is calculated by subtracting the average gene expression across control replicate samples at one time point from the average gene expression across NNV-infected replicates at the same time point after previous log_2_ transformation of the whole expression dataset. Please note that each heatmap is expressed on a log_2_ scale. Heatmaps are associated with a presence/absence heatmap depicting the membership of each gene to a specific pathway(s) with a purple square. Pathway names are reported at the bottom of the heatmap. (**C**) Average standardised expression across all significant genes of the “core ISG” in brain (circles) and head kidney (triangles) in each time point. Symbols depict the mean ± SD expression across all significant genes and are joined by the segments. Horizontal dashed line marks the expression level 0.

We tested the specific involvement of the type I interferon (IFN) response by using a “core” list of Interferon Stimulated Genes (ISGs), which has been identified by Shaw et al. ^39^ as consistently conserved across several vertebrate orders. In Br, a great proportion of this “core vertebrate ISGs” (i.e. 39 genes out of 62) showed an increased expression with time (Fig. 4A, Suppl. Table 7), including the antiviral genes *MX1* and *RSAD2* (known as Viperin) and two key RNA sensors (dhx58/lpg2 and ifih1/mad5^40^). The same gene set had a significant temporal pattern also in Hk (Fig. 4B, Suppl. Table 8), but comprised less genes in comparison to Br (i.e. 18 genes out of 62). In both tissues, all the components of the transcription factor IFN-stimulated gene factor 3 (ISGF3), i.e. *STAT1* and *STAT2* and *IRF9* showed a significant up-regulation from 24/48 hpi, although IRF9 in Br had a lower degree of up-regulation in comparison to the other two genes. Interestingly, between the two tissues the temporal trends of average gene expression for ISGs were quite different (Fig. 4C). An up-regulation pattern started to appear at 24 hpi in Hk, later on in Br. Furthermore “core ISGs” were on average down-regulated at 12 hpi in Hk, while their expression was two and four times higher at 24 hpi and 48 hpi, respectively. The same set of genes in Br showed no substantial time-dependent pattern until 48 hpi, when their average expression increased about eight times in comparison to the previous time points.

**Fig. 5:**
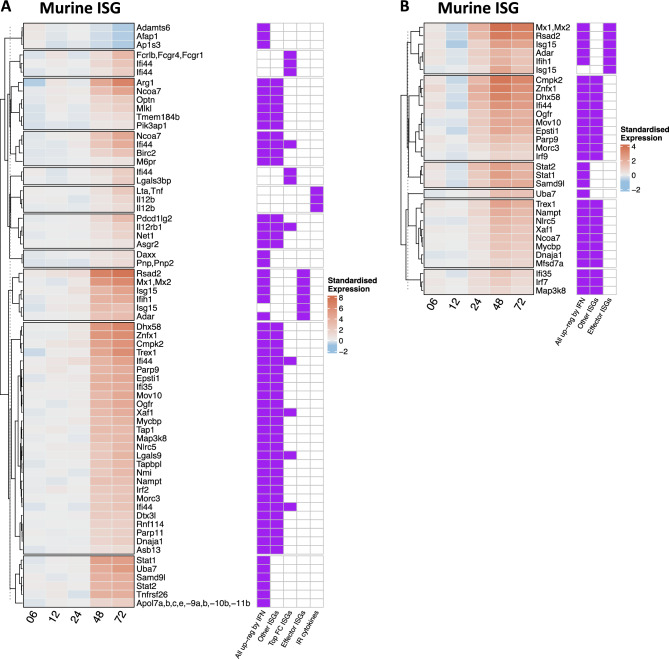
Heatmap of gene sets showing a significant time-dependent expression profile of the “Murine ISG” gene set in (**A**) brain tissue and (**B**) head kidney. The standardised expression is calculated by subtracting the average gene expression across control replicate samples at one time point from the average gene expression across NNV-infected replicates at the same time point after previous log_2_ transformation of the whole expression dataset. Please note that each heatmap is expressed on a log_2_ scale. Heatmaps are associated with a presence/absence heatmap depicting the membership of each gene to a specific pathway(s) with a purple square. Pathway names are reported at the bottom of the heatmap.

An involvement of the IFN response following NNV infection was further confirmed by testing an additional gene set from Goczi, et al. ^41^, in which effector ISGs were identified in a murine dendritic cell line stimulated with poly I:C. In a similar fashion to what was observed for the “core ISG” in the present study, a high number of these genes displayed a significant temporal pattern (Fig. 5, Suppl. Tables 7 and 8) with increasing expression levels with time in infected animals and a higher number of genes was found in Br in comparison with Hk. Among those, effector ISGs such as the antiviral genes *Mx1*, *Rsad2* and *Adar,* which play key roles in innate antiviral immunity, and galectin 9 (*Lgals9),* were found.

Finally, three putative interferon induced genes, all encoding a copy of interferon alpha inducible protein 27-like 2A, were significantly up-regulated in both tissues of infected fish at 48h and 72h. As already mentioned, these genes are located with the narrow high confidence region for a major QTL conferring higher resistance to VNN in sea bass^21^.

### Detection of viral reads

Reads that did not map to the sea bass genome were used as input by the tool VIRTUS2 and mapped against a database of 1894 viral genomes to search for NNV reads (Suppl. Fig. 3, Suppl. Table 9). In Br, the ratio of viral reads over sea bass reads showed a significant increase at 48 and 72 hpi only for reads mapping the RNA2 of the virus. In Hk, the we did not observe an increase in the ratio of viral reads over sea bass reads at any time point.

## Discussion

Infection by NNV possibly represents the major viral threat for farmed marine fish considering the broad host spectrum and the high mortalities reported upon infection. Several species, however, show complete or partial resistance to VNN, while genetic variation in resistance has been reported in susceptible species^42,43^. The molecular mechanisms underlying the diverse level of susceptibility observed between and within teleost species, unfortunately, remain rather elusive. Recently, host factors potentially involved in viral attachment and entry have been reported for the red spotted grouper^44^, although these results await validation in other species. Comparative transcriptome analysis of host response to NNV might provide evidence for divergent immune responses across teleost taxa. In fact, by comparing the robust IFN response in the gilthead sea bream, which is substantially resistant to VNN and the apparent low-absent IFN-mediated immune activation in the European sea bass, a highly susceptible species, it has been proposed that the differential IFN activation of IFN cascade might explain the markedly different degree of susceptibility between these species^7^. To fully evaluate the immune response to NNV at the transcriptome level, however, it is crucial to address its temporal profile. Time course RNA-seq analysis with sufficient time points and across key tissues is therefore essential to understand how gene expression dynamically changes, specifically during the initial stages of the infection (i.e. up to 72 hpi). Here, we show that gene networks specific for the nervous system (brain, pituitary, retina) are rapidly and progressively down-regulated likely impairing its normal functioning. In parallel, from 24 hpi onward, we found a significant impairment of the normal functioning of platelets, with implications associated with normal blood coagulation, which may lead to congestions of the blood vessels evolving also to haemorrhages, as documented by Toffan and Panzarin ^45^ via histological examinations. Concomitantly, in the head-kidney, we detected a significant peak in carbohydrate metabolism at 12 hpi. While this might suggest the onset of a Warburg effect possibly triggered by NNV, as reported for other viruses (e.g.^46,47^), our evidence clearly indicate that this effect does not last. In subsequent time points a remarkable down-regulation of carbohydrate metabolism pathways was observed, in agreement with Huang, et al. ^48^, who reported that NNV induces initially a Warburg effect but then, later during the infection, NNV induces fatty acid synthesis to promote its own replication. An intriguing finding from our GO analysis was the significant enrichment of genes associated with the ‘Neuroinflammation’ pathway among the upregulated genes in the brain tissue at later time points post-infection (48–72 hpi). This neuroinflammatory signature aligns with previous studies demonstrating the ability of NNV to induce inflammatory responses in the central nervous system of infected hosts^49,50^. The upregulation of neuroinflammatory genes may contribute to the neuropathological changes and neurological symptoms characteristic of NNV infection, such as abnormal swimming behavior and extensive brain damage. Neuroinflammation could potentially exacerbate tissue injury and disease progression by triggering excessive inflammatory responses and neurodegeneration. Further investigation into the specific neuroinflammatory mechanisms induced by NNV and their role in viral pathogenesis is warranted, as it may provide insights into potential therapeutic targets for modulating inflammatory processes and mitigating neuropathological consequences.

### Onset of IFN response is tissue and time-dependent

The transcriptome profiles of both tissues suggest a significant inflammatory and immune response, with a similar onset timing at 48h, but brain showed a much larger number of significantly up-regulated genes. By using targeted gene sets based on available evidence of the conserved core of IFN response, we demonstrated a clear and significant immune response in sea bass infected with NNV, and in particular of the IFN pathway. Of the 62 ISG that compose the “core vertebrate ISGs” identified as always up-regulated by IFN in 10 different vertebrate species by Shaw et al. ^39^, 39 genes (~ 63%) showed a significant temporal pattern of expression in the brain tissue of infected sea bass while 18 were found in head kidney. In a similar fashion, the majority of the ISGs reported by Goczi et al. ^41^ following poly I:C injection, a molecule that is structurally similar to dsRNA, were strongly up-regulated in brain and head kidney of infected sea bass, especially after 48 hpi, suggesting a general induction of IFN-induced antiviral program upon infection. Such evidence is in agreement with reports from other studies^6,7,14^, but it is quite at variance with the modest immune activation reported by Lama et al. ^15^, in NNV-infected sea bass. However, it is possible that the different size of experimental animals used or the viral dose adopted, may have contributed to the different response observed between the two studies. In fact, mortality rates between this work and Lama et al. ^15^ showed some differences (i.e. overall mortality rates were ~ 46% and ~ 90%, respectively).

A major key component of the type-I IFN signaling cascade is constituted by the activated transcription factor ISGF3, which forms upon association of STAT1, STAT2 and IRF9 and then translocates to the nucleus to induce transcription by binding IFN-stimulated response elements^18,19,51^. The genes composing this transcription factor are known to be responsive to NNV infection, as demonstrated recently in gilthead sea bream^7^. In our infected seabass, the up-regulation of all components of ISGF3 confirms our hypothesis of an activation of IFN pathway via activation of this transcription factor following NNV infection from 24 hpi, which is further supported by the significant number of genes of the ISG gene sets being differentially expressed from 24hpi onward (Figs. 4, 5).

### Comparative assessment of time-dependent onset of IFN response in various teleosts

In the head kidney of *Oryzias latipes* and *Lates calcarifer* infected with NNV, the up-regulation of *IFNa* or *IFNd* becomes evident 12 hpi^52,53^ and only 12/24 h later the IFN-mediated response is visible via the activation of *ISG15*, *ISG56* and *Mx*^52^. These results suggest that, despite the possible differences between various species, the onset time of the immune response has a delay of about 36 h from the initial infection, in agreement with the results showed here. Some studies have used the expression of the antiviral genes Mx as reporters for the activation of type I IFN^6,10^ and found an up-regulation of these genes after 24 or 48 h post infection in the European sea bass, depending on the study. Our results are concordant with these findings; in fact, the *Mx1* was significantly up-regulated from 24 hpi in Hk while its up-regulation became apparent only from 48 hpi in Br (Fig. 4). The fact that this gene was not up-regulated earlier during the infection could confirm the hypothesis proposed by Carballo, et al. ^6^, who stated that the European sea bass IFN system requires a high viral load before it can be induced, thus explaining the late onset of Mx up-regulation.

Further, additional antiviral and PAMP sensing genes among the “core ISG” gene set shared the same temporal pattern of the Mx1 gene. Among them, *RSAD2* (also known as *Viperin*) which is an ISG effector suppressing viral replication^19^. Together with these antivirals and PAMP sensing genes, an up-regulation of the IFN suppressors *NMI* and *IFI35* was also observed from 48 hpi. Higher expression of IFN suppressors may be either part of an avoidance mechanism of the RGNNV virus to elude the host response or a physiological mechanism to prevent hyper-activation of the IFN response, also in light of the extremely high levels of expression of some effector genes. Excessive inflammation induced by unbalanced IFN response may damage tissues, leading to neurodegeneration in the inflamed brain, as stated by Pereiro, et al. ^7^.

### Time-dependent modulation of genes belonging to HPI axis

Stress response in fish following viral infection is still poorly characterized. Here, a more complex, time-dependent modulation of the genes belonging to the HPI axis was observed compared to what reported by Lama et al. ^15^. In that work, only two time points were analysed, (i.e. 24 hpi and 72 hpi), showing that many HPI genes were up-regulated at 24 hpi and down-regulated at 72 hpi. In the present study, a modest up-regulation was found between 6 and 24 hpi, with a peak at 12 hpi in the brain. In the head kidney, this change was not coupled by a significant up-regulation of the genes involved in the production of cortisol (data not shown), the main stress response effector, at variance with Lama et al. ^15^. At 48 hpi our data showed a strong down-regulation, followed by a weak up-regulation at 72 hpi, indicating a second shift in the expression of these genes regulating the HPI axis. As already mentioned, partial discordance between the two studies could be explained by different experimental set-ups, although it seems evident that a higher number of time points allows a better understanding of temporal patterns in host response.

### Involvement of macrophages in response to NNV infection

The innate-branch of the immune system is essential for early host response, and macrophages are among the key components of this system. Our analysis confirmed that macrophages are significantly recruited in sea bass response to NNV, since multiple genes linked to macrophage activation, migration, and chemotaxis showed a significant temporal profile, with increasing expression levels over the course of infection. This corroborates the idea that an infiltration of these cells as well as other types of lymphocytes takes place in sea bass brain during the infection, as recently proved by Pianese et al. ^54^, as well as by other authors in other NNV infected fish^7,16,55,56^, although their involvement in the damage to the nervous tissue caused by the virus attack remains to be ascertained.

## Conclusions

The process of infection is dynamic, as host responses change in time (e.g. ^32,46,57,58^), hence a time course approach constitutes an essential tool to shed light on the pathogenesis of infectious diseases. By applying this approach, we have found a robust immune response triggered by IFN in the European sea bass following injection with NNV that became particularly evident from 48 hpi, thus clarifying the involvement of this antiviral cytokine during NNV infection in this species. In this respect, it seems that the European sea bass is highly susceptible to VNN “despite” the presence of a robust IFN activation rather than because of its modest-absent IFN-mediate response as proposed by Lama et al. ^15^. It is possible that subtler differences in innate immune response are the result of either activation of different subsets of genes, or different timing and tissue specificity of IFN-mediated response. For instance, Pereiro et al. ^7^ reported IFN pathway activation already at 24 hpi and a stronger response at 72 hpi in the brain of gilthead sea bream, a species mostly resistant to NNV. In the head kidney, the trend was reversed, with a great induction of innate immune response at 24 hpi and a less marked one at 72 hpi. The latter evidence is at variance with what reported in the present study for the European sea bass, where response in the head kidney at the gene expression level was substantially less pronounced that the one in the brain, both starting at 48 hpi. A more rigorous comparison would require, however, more time points for the response in the gilthead sea bream. Unfortunately, so far, only a handful number of studies has included more than one time point in their experimental work, e.g.^7,14,15^ and none has formally included this factor in their analyses. While the “classical” approach with pairwise comparisons has been extremely useful in understanding the specific changes that happen at a certain stage of the infection (e.g. in the early stages of the infection), this approach is limited in providing an overview of the changes that occur throughout the infection. In the light of the drop of the costs for sequencing that we have experienced in the past years and the introduction of kits allowing to reduce even further the costs (e.g. the 3’ tag sequencing kits), it is now becoming easier to sequence samples from multiple time points. For these reasons we advocate that time course analysis should be applied in such studies to help understanding how the host is responding to the invading virus.

## Supplementary Information

Supplementary Figures.

Supplementary Tables.

## Data Availability

The sequences obtained are available in NCBI Sequence Read Archive BioProject PRJNA1200971).
