# Supplementary material for: Temporal profiling of host transcriptome highlights time- and tissue-dependent Interferon pathway activation in NNV-infected European sea bass
Source: Sci Rep. 2025 Jul 10;15:24941. doi: 10.1038/s41598-025-09705-5 (PMC12246263; doi:10.1038/s41598-025-09705-5)
Supplement: Supplementary file 1 — Supplementary Figures. [file 41598_2025_9705_MOESM1_ESM.docx]

# Title:

**Temporal profiling of host transcriptome highlights time- and tissue-dependent Interferon pathway activation in NNV-infected European sea bass**

# Authors:

Luca Peruzza^1^^, Giulia Dalla Rovere^1^, Serena Ferraresso^1^, Rafaella Franch^1^, Daniela Bertotto^1^, Francesco Pascoli^2^, Gaia Bacchin^2^, Anna Toffan^2^, Luca Bargelloni^1^

^1^ Department of Comparative Biomedicine and Food Science, University of Padova, Viale dell’Università 16, 35020, Legnaro (PD)

^2^ National Reference Laboratory for fish diseases, Istituto Zooprofilattico Sperimentale delle Venezie, Viale dell’Università 10, 35020 Legnaro (PD)

^^^ Corresponding author: Luca Peruzza, [luca.peruzza@unipd.it](mailto:luca.peruzza@unipd.it)

# Supplementary Figures:

**Suppl. Figure 1:** Principal Component Analysis of RNAseq libraries sampled from: **A)** Brain and **B)** Head kidney tissues. Each dot represents a biological replicate. Colors refer to hours post injection (i.e. 6, 12, 24, 48 and 72 hpi) while shapes refer to the two conditions (i.e. Mock-injected, circles; NNV-injected, triangles).

**Suppl. Figure 2:** Heatmap of gene sets showing a significant time-dependent expression profile of the “Immune cell atlas” gene set in **A)** brain tissue and **B)** head kidney. The standardised expression is calculated by subtracting the average gene expression across control replicate samples at one time point from the average gene expression across NNV-infected replicates at the same time point after previous log_2_ transformation of the whole expression dataset. Please note that each heatmap is expressed on a log_2_ scale. Heatmaps are associated with a presence/absence heatmap depicting the membership of each gene to a specific pathway(s) with a purple square. Pathway names are reported at the bottom of the heatmap.

**Suppl. Figure 3:** NNV reads detected in samples at different time points. Symbols depict the average ± SD of the rate hit, defined as the number of viral reads mapped to the NNV genome over the number of reads mapped to the European sea bass, in control (circles) or NNV-infected (triangles) samples. Colors refer to the two parts of the NNV genome, RNA1 and RNA2 respectively in green and red.
